# Sacrospinous ligament suspension with transobturator mesh *versus* sacral colpopexy for genital prolapse

**DOI:** 10.6061/clinics/2016(09)01

**Published:** 2016-09

**Authors:** Cássia R.T. Juliato, Maira F.G. Mazzer, Juliana M Diniz, Catarina H.S. Farias, Edilson B de Castro

**Affiliations:** Universidade de Campinas, Departamento de Ginecologia, Campinas/SP, Brazil

**Keywords:** Vaginal Prolapse, Surgical Mesh, Pelvic Organ Prolapse, Efficacy, Recurrence

## Abstract

**OBJECTIVE:**

To compare the safety and efficacy of abdominal sacral colpopexy and sacrospinous ligament suspension with the use of vaginal mesh for apical prolapse.

**METHOD:**

This retrospective study was conducted from 2005 to 2012 and included 89 women with apical prolapse who underwent surgery. Assessments included pre- and postoperative Pelvic Organ Prolapse Quantification (POP-Q) stage. Rates of objective cure and immediate/late complications were compared.

**RESULTS:**

In total, 41 of the 89 women underwent sacrospinous ligament suspension, and 48 of the women underwent abdominal sacral colpopexy. A total of 40.4% of the women had vault prolapse (*p*=0.9361). Most of them had no complications (93.2%) (*p*=0.9418). Approximately 30% of the women had late complications; local pain was the main symptom and was found only in women who underwent the abdominal procedure (25.6%) (*p*=0.001). Only the women who were submitted to the vaginal procedure had mesh exposure (18.4%). The objective success rate and the rate of anterior vaginal prolapse (*p*=0.2970) were similar for both techniques.

**CONCLUSION:**

Sacrospinous ligament suspension was as effective and had a similar objective success rate as abdominal sacral colpopexy for the treatment of apical prolapse. Sacrospinous ligament suspension performed with the use of vaginal mesh in the anterior compartment was effective in preventing anterior vaginal prolapse after surgery.

## INTRODUCTION

Several surgical techniques can be used to treat apical prolapse. These include sacrospinous ligament fixation via the vagina and abdominal colpofixation to the sacral promontory (i.e., sacral colpopexy) 1,2.

Sacrospinous ligament fixation requires less surgical time and provides faster recovery than the alternatives, however, it also has a greater association with the onset of postoperative cystocele, which can be understood by the female patient as a failure of the technique 3,4. One method to minimize prolapse of the anterior wall (cystocele) after fixation to the sacrospinous ligament is the use of synthetic non-absorbable material (i.e., meshes) during surgery. Repairing a cystocele with non-absorbable polypropylene material is better than the traditional colporrhaphy and leads to a lower recurrence rate of anterior vaginal wall prolapse 3.

However, the use of meshes is not free of risks or complications, the main risks being extrusion and dyspareunia 5. The International Urogynecological Association (IUGA) accepts the use of meshes in women who will undergo sacrospinous fixation, as these women generally have advanced stage prolapse or apical prolapse, and the benefits outweigh the risks under these conditions 6. The use of mesh in the anterior wall can also be employed for women undergoing sacrospinous ligament fixation in order to decrease the rate of anterior wall prolapse following the procedure. However, few studies have examined this association.

The objective of the current study was to compare the efficacy and safety of vaginal fixation to the sacrospinous ligament using mesh in the anterior wall and sacral colpopexy for the correction of apical prolapse. This comparison was made by analyzing the objective cure, complication and failure rates after each procedure.

## MATERIALS AND METHODS

A retrospective design was used to evaluate the medical records of 89 women who underwent surgery for genital apical prolapse repair, 48 of whom underwent abdominal sacral colpopexy and 41 of whom underwent sacrospinous ligament fixation with mesh placement on the anterior wall of the vagina. All procedures were performed at the Department of Obstetrics and Gynecology, School of Medical Sciences, University of Campinas (Unicamp), from 2005 to 2012. This study was approved by the Ethics Committee of the Faculty of Medical Sciences under number 152451113.8.00005404 on 08/08/2013.

The two standard procedures used for apical prolapse at the hospital were abdominal sacral colpopexy and sacrospinous ligament fixation without anterior wall correction. The women were informed about the two procedures and were free to choose which one they preferred. All surgeries were performed by the same operator (20 years of experience, more than 5000 surgeries). The techniques are described below.

Sacrospinous ligament fixation with mesh placement on the anterior wall with a double transobturator pass.

All of the women who had a uterus underwent vaginal hysterectomy. After opening the posterior vaginal mucosa, two stitches with Vicryl thread were passed bilaterally through the sacrospinous ligament using direct visualization, followed by fixing of the stitches in the vaginal vault. Then, a longitudinal opening was made in the anterior vaginal mucosa. Dissection of the vesico-vaginal fascia was performed bilaterally up to both tendon arches.

Gynecare Prolift^TM^ mesh kits (Johnson & Johnson Company, Somerville, New Jersey, USA) were used. The needles from a mesh kit were placed bilaterally along the superomedial rim of the transobturator foramen, and the upper extension arms of the mesh were fixed to each side of the needles with externalization up to the entry point of the genitofemoral fold. An additional two needles were placed bilaterally through the inferomedial rim of the transobturator foramen (with a 2 cm lateral entrance hole and at 2 cm under the genitofemoral fold at the height of the external urethral meatus; the exit path was through a hole in the anterior vaginal mucosa). The lower extension arms of the mesh were placed on each side of the needles, with an exit path that reached up to the entrance point of the genitofemoral fold. The upper rim of the mesh was placed on the vesico-vaginal fascia just below the bladder neck using 0 or 1 Polyglactin 910 thread. The lower rim was placed on the vaginal vault parametria using two Prolene 2.0 stitches. After this procedure, the anterior vaginal mucosa was closed with 910 Polyglactin 0 threads. The vault was fixed using knots on the sacrospinous ligament. The mesh was adjusted without tension. The mesh extension arms were cut close to the skin, which was closed with catgut 2.0. The mesh used was a polypropylene, macroporous and monofilament mesh.

### Abdominal sacral colpopexy

The skin was incised up to the Pfannenstiel or infraumbilical median if a woman presented with this type of prior incision. All of the women who had a uterus underwent abdominal hysterectomy. The sacral retroperitoneum was opened to reveal the promontory periosteum. Mesh was placed between the sacral promontory and the vaginal vault, correcting anterior and posterior defects when present. The fixation of the mesh on the promontory periosteum and vault was performed using Prolene 0. After fixation, the mesh was covered with the parietal peritoneum so that the mesh would not be exposed in the abdominal cavity. The pelvic wall was then closed in planes. There were no anterior wall vaginal corrections in the abdominal sacral colpopexy group. Women with posterior prolapse underwent vaginal wall reconstruction.

The evaluation of a prolapse was conducted using the Quantification System of Pelvic Organ Prolapse (POP-Q). Study data were collected using an elaborate form that included dependent variables such as efficacy (defined by objective cure and failure rates), safety (defined by immediate complications such as intraoperative bleeding and infection), and late complications (occurring within 6 weeks after surgery) such as pain, mesh extrusion, infection, vaginal bleeding, vaginal discharge, fistulas and dyspareunia. The control variables included degree of uterine prolapse, age, parity, race, body mass index (BMI), comorbidities, previous surgeries, smoking status and use of hormone replacement therapy. Objective cure was defined as the absence of a POP-Q stage prolapse less than or equal to 2. The first post-operative review was performed 6 weeks after surgery and 6 follow-up visits were conducted over 6 months.

The points from the POP-Q (7,8) were assessed during the first visit and at the post-operative review. The POP-Q was applied before surgery and at a later revaluation. Genital prolapse was assessed qualitatively in stages and quantitatively in centimeters. Cure, complication and recurrence rates were evaluated through simple prevalence and were compared using the chi-square test or Fisher's exact test. For non-parametric variables, the Mann-Whitney test was used. For the variables that were evaluated during follow-up, the Wilcoxon paired test for POP-Q measurements was used. The significance level was 5%, and SAS was used for data analysis was SAS.

## RESULTS

The median follow-up was 9 months for women who underwent sacrospinous ligament fixation and 6 months for the women who sacral colpopexy. The average age was 63.1 (±8.7) years for the women who underwent sacrospinous ligament fixation and 63 (±8.4) years for those who underwent sacral colpopexy, with no significant difference between the two groups (*p*=0.8373). In relation to the women included in the study, 40.4% had vault prolapse, 19% had previously underwent abdominal hysterectomy and 21.4% had previously underwent vaginal hysterectomy. There were no differences in these variables between the two groups (Table 1).

Regarding the POP-Q classification system used for prolapse prior to surgery, most of the women (58.4%) had stage 3 apical prolapse (Table 2). In assessing the presence of anterior wall prolapse prior to surgery, it was noted that there were differences between the two study groups (*p*<0.0001).

Regarding prolapse stage after surgery, there was no difference between the groups regarding the presence of prolapse in the anterior wall. When evaluating apical prolapse, the authors found that there were 3 cases of failure in the women who had underwent sacrospinous ligament fixation. Considering the objective cure rate as less than or equal to stage 2 apical prolapse, the cure rate was 95.8%, with no difference between the groups (90.9% in the group who underwent sacrospinous ligament fixation and 100% in the group who underwent sacral colpopexy). There were only three cases of failure. All were in the group who underwent sacrospinous ligament fixation, and the average follow-up was 11.1 months (Table 2).

In analyzing the immediate complications, the vast majority of the cases that underwent surgery presented no complications (93.2%), with no difference between the groups (*p*=0.9418). The most frequent complications were increased bleeding and a need for transfusion, with no difference between the groups (Table 3). There were no vascular, intestinal or urinary tract injuries.

Approximately 30% of the women had late complications (after 40 days). The most common complication was local pain, which was present only in the women who underwent the abdominal technique (25.6%) (*p*=0.001). Mesh exposure occurred in 9.1% of the total cohort: 18.4% of the women who underwent the vaginal procedure had mesh exposure, and none of the women who underwent the abdominal procedure had mesh exposure. This difference was significant between the groups (*p*=0.0052). Only 2 of the women (4.9%) who underwent sacral colpopexy presented with vaginal discharge (*p*=0.4948).

Most of the women did not experience postoperative apical prolapse (91% of the women who underwent colpofixation to the sacrospinous and 78.9% of the women who underwent sacral colpopexy did not have this complication). Regarding anterior wall prolapse, 9% of the women who underwent vaginal surgery had stage 3 prolapse (Table 3).

**Figure 1:**
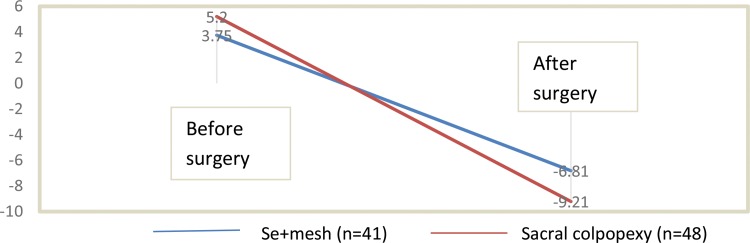
Comparison of apical prolapse before and after surgery. Apical prolapse, point C, POP-Q classification. Se+mesh=sacrospinous ligament suspension with transobturator mesh.

When analyzing the POP-Q points, the authors observed a significant improvement in the Aa and Ba points in the anterior wall in both surgeries, with no difference between them (Table 4). The average point C (point of apical prolapse) showed significant improvement in both of the surgical groups operated (Table 4 and Figure 1). Vaginal size after surgery was larger in the group who underwent the abdominal technique. The average vaginal size was 9.31 cm in the women who underwent sacral colpopexy and 8.15 cm in the vaginal surgery group (*p*=0.0174). However, there was no difference between the groups when considering the difference between the preoperative and postoperative values, which leads to the conclusion that there was no difference regarding vaginal size between the two surgeries (Table 4).

## DISCUSSION

Genital prolapse can affect a woman’s quality of life, impacing the psychological, social and financial aspects of her life 9. With the aging of the population, it is estimated that the number of cases of genital prolapse in the United States will double in the next 30 years 10. Anterior genital prolapse is the most prevalent; however, apical prolapse normally progresses with prolapses beyond the hymen and is therefore more symptomatic. Furthermore, providing apical support has an important role in sustaining the anterior wall because if insufficient support is provided, treatment to correct the anterior and posterior walls may be ineffective 11.

The biggest failure in sacrospinous ligament fixation is anterior wall prolapse, probably due to the posterior deviation of the vaginal axis 4,12,13. In a previous study conducted using this technique, an anterior wall prolapse rate of 39.7% (12) was obtained. The literature shows an anterior wall prolapse rate ranging from 17.3 to 25.3% (4). In contrast, in the current study, a low anterior prolapse rate was found, with no difference between the two techniques used. This is likely due to the use of mesh in the anterior wall during sacrospinous ligament fixation. The superiority of using synthetic meshes has been well described in the literature 14-16. One study of the use of synthetic mesh via a double pass on the transobturator foramen also had satisfactory results with regard to anterior wall prolapse 17.

This study evaluated two techniques for the treatment of apical prolapse (uterine or vault): an abdominal technique that used fixation to the sacral promontory and a vaginal technique that used fixation of the vault to the sacrospinous ligament by employing vaginal mesh. The objective success rates were satisfactory for both types of surgery, and there were no differences in their anterior vaginal prolapse rates. Sacral colpopexy has been accepted as the gold standard for apical prolapse treatment. In a systematic review, the objective success rate of sacral colpopexy was greater than that for fixation to the sacrospinous ligament. The current study showed a similar objective cure rate for the two techniques, which confirms that both surgeries are effective for apical prolapse treatment.

Several kits have been developed to treat prolapses of several compartments; some of these kits try to avoid the transobturatorial passage 18-20. The placement of needles on the transobturator foramen can cause injuries such as vascular and nerve damage, but it provides better mounting of mesh on the anterior wall and possibly better anatomical results. In the present study, there were no serious complications and both techniques were deemed safe. There were no vascular lesions or instances of organ damage. Vascular lesions and bleeding are rare events 21. Some reports have suggested that lesions are more frequent in sacral colpopexy surgery 22, but this finding was not observed in our study because we had only two cases that required transfusion (one in each group).

Mesh use in prolapse surgery has received criticism due to the potential for adverse events related to its use. The most frequent adverse event reported is extrusion, which may lead to important disorders for cases with extrusion to an organ. The literature reports an extrusion, erosion or exposure rate of up to 29% with the use of vaginal mesh 23-25. The exposure rate in the current study was lower than those reported in the literature for women undergoing vaginal procedures. All the exposure cases were in the vaginal mucosa and were small, avoiding the need for surgical intervention. Recently, the IUGA evaluated mesh erosion and showed that in asymptomatic women treatment is usually conservative 26.

Another complication associated with the use of vaginal meshes is the presence of vaginal discharge. A study of women diagnosed with mesh extrusion noted that 30.9% complained of vaginal discharge. This confirms that the use of mesh may increase the incidence of vaginal discharge. However, in our current study, few women presented with vaginal discharge after surgery and all of them were in the group that underwent sacrospinous ligament fixation 27.

The use of the above vaginal technique has been consistently associated with diminished vaginal length after surgery, which could result in dyspareunia and sexual dysfunction. One study that compared women who underwent sacrospinous ligament fixation and sacral colpopexy with controls (women) concluded that if increased vaginal length is desirable, sacral colpopexy is more appropriate 28. In the current study, however, no difference was noted between surgeries for vaginal shortening.

One limitation of the current study is that the women who underwent surgery were not homogenous with regard to prolapse stage prior to surgery. The women who underwent sacral colpopexy presented more advanced stages and thus had more serious conditions than those who underwent sacrospinous ligament fixation. This might explain why there was no difference in the success rates of the two surgeries. Another limitation of the study was the use of medical records, as there were many missing and unknown data. The strengths of this study include the decrease in anterior wall prolapse rate following the use of mesh in the anterior wall, which improved the results and decreased the overall failure rate.

In conclusion, sacrospinous ligament fixation and sacral colpopexy are effective and safe techniques for the treatment of apical genital prolapse, with similar cure rates for the apical compartment. Sacrospinous ligament fixation using mesh in the anterior vaginal wall showed cure and relapse rates in the anterior compartment that were similar to those found in women undergoing the abdominal technique. There were no serious complications, including those related to the use of mesh, in either operation.

## AUTHOR CONTRIBUTIONS

Juliato CR was responsible for the project development and manuscript writing. Mazzer MF and Castro EB were responsible for the manuscript writing. Diniz JM and Farias CH were responsible for the project development and data collection.

## Figures and Tables

**Table 1:** Characteristics and types of surgery.

		Surgery					
	TOTAL		Sacrospinous		Sacral Colpopexy		*p*
	n	%	n	%	N	%	
**Race**							0.0288
White	74	83.1	30	73.2	44	91.7	
Black	1	1.1	1	2.4	0	0.0	
Brown	14	15.7	10	24.4	4	8.3	
**Gestation**							0.0063
0	2	2.2	0	0.0	2	4.2	
1	1	1.1	0	0.0	1	2.1	
2	19	21.3	4	9.8	15	31.3	
3 or more	67	75.3	37	90.2	30	62.5	
**Parturition**							0.0155
0	2	2.2	0	0.0	2	4.2	
1	3	3.4	0	0.0	3	6.3	
2	21	23.6	6	14.6	15	31.3	
3 or more	63	70.8	35	85.4	28	58.3	
**BMI**							0.3365*
Unknown	8		4		4		
Normal	26	32.1	14	37.8	12	27.3	
Obesity	24	29.6	12	32.4	12	27.3	
Overweight	31	38.3	11	29.7	20	45.5	
**Tabagism**							0.7467
Unknown	4		3		1		
No	75	88.2	33	86.8	42	89.4	
Yes	10	11.8	5	13.2	5	10.6	
**Comorbidities**							0.5217*
Unknown	1		0		1		
No	25	28.4	13	31.7	12	25.5	
Yes	63	71.6	28	68.3	35	74.5	
**Abdominal hysterectomy**							0.9361*
Unknown	5		5		0		
No	68	81	29	80.6	39	81.3	
Yes	16	19	7	19.4	9	18.8	
**Vaginal hysterectomy**							0.7011*
Unknown	5		5		0		
No	66	78.6	29	80.6	37	77.1	
Yes	18	21.4	7	19.4	11	22.9	

Chi square/* Fisherߣs exact.

BMI Bone Mass Index.

**Table 2:** Grades of apical prolapse before and after surgery and cure rates.

		Surgery					
	TOTAL		Sacrospinous		Sacral Colpopexy		*p*-value
	n	%	n	%	n	%	
**Anterior stage before surgery**							< 0.0001
unknown	5		1		4		
0	1	1.2	1	2.5	0	0	
II	7	8.3	0	0	7	15.9	
III	63	75	37	92.5	26	59.1	
IV	13	15.5	2	5	11	25	
**Posterior stage before surgery**							0.002
unknown	10		5		5		
0	17	21.5	6	16.7	11	25.6	
I	4	5.1	3	8.3	1	2.3	
II	4	5.1	0	0	4	9.3	
III	43	54.4	26	72.2	17	39.5	
IV	11	13.9	1	2.8	10	23.3	
**Apical stage before surgery**							0.0034
II	20	22.7	15	36.6	5	10.6	
III	48	54.5	20	48.8	28	59.6	
IV	17	19.3	4	9.8	13	27.7	
**Anterior stage after surgery**							0.2970*
unknown	18		8		10		
0	53	74.6	23	69.7	30	7.9	
I	3	4.2	1	3	2	5.3	
II	12	16.9	6	18.2	6	15.8	
III	3	4.2	3	9.1	0	0	
**Posterior stage after surgery**							0.0454*
unknown	17		8		9		
0	59	81.9	26	78.8	33	84.6	
I	4	5.6	0	0	4	10.3	
II	7	9.7	5	15.2	2	5.1	
III	2	2.8	2	6.1	0	0	
**Apical stage after surgery**							0.0477*
unknown	18		8		10		
0	66	93	30	90.9	36	94.7	
I	2	2.8	0	0	2	5.3	
III	3	4.2	3	9.1	0	0	
**Objective impression**							0.0955
unknown	18		8		10		
Cured	68	95.8	30	90.9	38	100	
non cured	3	4.2	3	9.1	0	0	

Chi square/* Fisher’s exact.

**Table 3:** Immediate and late complications after surgery.

		Surgery					
	Total		Sacrospinous		Sacral Colpopexy		*p*
	n	%	n	%	n	%	
**Immediate complication**							0.9418
Infection	1		0		1		
None	82	93.2	38	92.7	44	93.6	
Other	1	1.1	1	2.4	0	0.0	
Blood loss	3	3.4	1	2.4	2	4.3	
Transfusion	2	2.3	1	2.4	1	2.1	
**Late complications**							0.8614*
Unknown	12		3		9		
No	54	70.1	27	71.1	27	69.2	
Yes	23	29.9	11	28.9	12	30.8	
**Infection**							1.0000
Unknown	12		3		9		
No	73	94.8	36	94.7	37	94.9	
Yes	4	5.2	2	5.3	2	5.1	
**Local pain**							0.0010
Unknown	12		3		9		
No	67	87.0	38	100.0	29	74.4	
Yes	10	13.0	0	0.0	10	25.6	
**Mesh exposure**							0.0866
Unknown	12		3		9		
No	68	88.3	31	81.6	37	94.9	
Yes	9	11.7	7	18.4	2	5.1	
**Blood loss**							0.4935*
Unknown	12		3		9		
No	75	97.4	38	100.0	37	94.9	
Yes	2	2.6	0	0.0	2	5.1	
**Vaginal discharge**							0.4948
Unknown	11		0		11		
No	76	97.4	39	95.1	37	100.0	
Yes	2	2.6	2	4.9	0	0.0	

Chi square/* Fisherߣs exact.

**Table 4:** Comparison of Pelvic Organ Prolapse Quantification points before and in the last follow-up visit after surgery.

POP-Q point (difference between before and after surgery)	Sacrospinous			Sacral Colpopexy			
	N	Mean	DP	n	Mean	DP	*p* value
Aa	25	-4.8	1.85	13	-3.46	2.15	0.0794
Ba	24	-7.79	2.43	13	-6.08	3.35	0.1369
C	25	-11	3.82	13	-12.69	5.01	**0.0459**
Total vaginal length	25	-1.28	1.93	15	-0.4	1.3	0.1587
Ap	25	-1.12	2.91	13	-1.46	2.11	0.8137
Bp	25	-4.52	4.52	13	-4.38	3.75	1.0000

*p* value: Mann-Whitney.
